# Editorial: Avian incubation conditions: Role in embryo development, physiology and adaptation to the post-hatch environment

**DOI:** 10.3389/fphys.2023.1130982

**Published:** 2023-01-13

**Authors:** Servet Yalçin, Edgar O. Oviedo-Rondón

**Affiliations:** ^1^ Department of Animal Science, Ege University, Izmir, Türkiye; ^2^ Prestage Department of Poultry Science, North Carolina State University, Raleigh, NC, United States

**Keywords:** incubation conditions, development, embryo, metabolism, poultry

## Introduction

This Research Topic on Avian Incubation Conditions is an inclusive treatise discussing the impact of several environmental factors during egg storage and in the incubators that may affect avian embryo and post-hatch development, physiology, metabolism, and growth. The objective of this project was to gather current relevant research on this topic, detect areas lacking knowledge or understanding, and propose methods of study to advance on embryology and avian incubation techniques. The impact of egg storage conditions, incubation temperature, hypoxia, moisture, and light, among other factors, were reviewed in this Research Topic. Embryo developmental and long-lasting effects of these factors were systematically revised. A total of 12 manuscripts were accepted for publication: 7 original research contributions; 3 reviews; one mini-review; and one paper related to methods.

Three papers presented recent findings related to egg storage effects on egg quality and embryo development. Egg storage is a common and necessary practice in poultry production to synchronize hatch, meet and regulate hatchling demands, and coordinate activities in the hatchery. However, prolonged storage and conditions during this period may significantly impact embryo survival, development, hatchability, and even life post-hatch. Minimizing these harmful effects has been a frequent Research Topic in avian incubation. Pokhrel et al. elucidated the molecular and cellular mechanisms involved in the better recovery of embryos during storage when eggs were exposed to 12°C rather than higher temperatures (18°C). These temperature-dependent mechanisms are related to the embryo’s transition from blastulation to gastrulation.

Studying the sequential effects of egg storage on egg quality and embryo development on the same eggs is challenging since most methods to evaluate egg quality are destructive. Adriaensen et al. used non-invasive tools such as computed tomography and magnetic resonance imaging in a study to evaluate the effects of egg storage duration on egg quality and embryo development. In this study, eggs were stored at 16°C and 80% relative humidity. These imaging technologies aided in visualizing and quantifying the negative impacts of egg storage on embryo development. Guinebretière et al. studied the effects of three storage temperature strategies (11.6°C or 18.3°C) together with pre-incubation in chicken eggs from young and old breeder flocks. The pre-incubation was applied on days 6 and 10 during the storage period of 14 days. Their results indicated that the low storage temperature (11.6°C) had similar results to warm egg storage (18.3°C) if pre-incubation is applied, and both treatments counterbalance the negative effects of prolonged egg storage on hatchability and chick quality regardless of breeder age; however, these authors did not detect differences among treatments on chicken live performance during rearing.

Original research and review manuscripts covered the impact of variation in temperature and other incubation factors on embryo development and long-lasting effects in the life post-hatch. Almeida et al. evaluated the effect of constant low (36°C), control (37.5°C), and high (39°C) machine temperature from day 13 onward on lipid metabolism, adipose tissue, and body composition. Their findings indicated that incubation temperature affects regional adiposity, lipid metabolism, and fat deposition in broilers.

Many studies in the past years have tried to determine the optimal embryo temperature during incubation, looking at diverse response parameters. Yalçin et al. reviewed current studies on this topic and the influence of temperature variation and light use during incubation. This review also included studies on cyclic temperature manipulations during critical periods of embryo development. Additionally, the review encompassed the potential impact of lighting on circadian rhythms vital for development and regulation related to improving the resistance of broilers to heat stress.

Tona et al. also reviewed the effects of incubation conditions such as temperature, relative humidity, turning, ventilation, *In ovo* feeding, and delay in feed access. These authors concluded that all these incubation factors might affect embryo parameters and post-hatch growth differentially according to exposure time and stage of development at the time of the stimulus in the incubator. The literature review presented by Wang et al. focused on the impact of temperature, humidity, oxygen density, ventilation, and lighting on the number, shape, and structure of embryo muscle fibers, with long-lasting effects on post-hatch muscle growth and meat quality. This paper also suggested future studies to evaluate the effects of incubation conditions on muscle cell regulation, proliferation, and meat quality looking for methods to improve the final poultry product.

Oxygen availability controls several mechanisms of development, tissue maturation, and cell metabolic regulation in avian embryos. The impact of hypoxia during incubation on the post-hatch performance of broilers subject to suboptimal environmental temperature was presented in original research conducted by Haron et al. Hypoxia for either 12 h (17% O_2_) during days 16–18 of incubation or continuous hypoxia for 48 h from days 16–17 were compared with incubation under normoxic conditions (21% O_2_). Hatchlings were raised under cold, hot, and diurnal cyclic temperatures. Broiler results, up to 42 days of age, indicated that hypoxia during that critical period of embryo development caused adaptive metabolic responses that improved thermoregulation, feed efficiency, and breast muscle growth. The authors proposed strategic hypoxia during incubation as a tool to adapt poultry to post-hatch suboptimal environmental conditions.

Relative humidity variability during incubation from 25% to 93% was evaluated by Branum et al. While hatchability, embryo dry body mass, and acid-base regulatory responses were not affected by this factor, incubator relative humidity caused significant differences in tissue water content and body mass at hatch. Embryo hydric balance can also be affected by egg treatments. Gregorich et al. induced moisture loss in leghorn and broiler eggs by drilling two 1.5 mm diameter holes in the eggshells. They observed the immunity cell response and transcription of interleukins in embryos and chicks facing a challenge with lipopolysaccharides. These researchers concluded that this double-hole treatment and faster moisture egg loss could reprogram embryo gene transcription to facilitate immunity cell survival and responses to an immunological challenge.

A mini-review by Sukparangsi et al. described the benefits of avian embryonic culture *In Ovo* and *In vitro* cell culture versus the traditional *Ex-Ovo* methods. However, the relationships among these methods are discussed. These methodologies can help better understand avian development mechanisms and unravel the transcriptional networks that regulate cell differentiation in embryos. In the same line of ideas related to methodologies of studying embryo development, Dave et al. proposed a novel methodology called the egg-in-cube system to study the avian blastoderm with intact tissue tensions on its native yolk. This technique could solve the common flaw of embryo culture techniques. This new methodology could allow researchers to explore fundamental questions in early embryogenesis on its native yolk.

In summary, the papers compiled on this Research Topic offer a complete overview of the impact of multiple factors that may affect various embryo developmental parameters during storage and incubation. It was evident in all papers that incubation conditions have consequences in the life post-hatch. Consequently, incubation conditions could be used to adapt the hatchlings to adverse environmental conditions or improve meat quality, immunity, and health. This Research Topic also includes reviews of methodologies to enhance the understanding of embryology and the impact of avian incubation factors.

